# Can the Error Detection Mechanism Benefit from Training the Working Memory? A Comparison between Dyslexics and Controls — An ERP Study

**DOI:** 10.1371/journal.pone.0007141

**Published:** 2009-09-25

**Authors:** Tzipi Horowitz- Kraus, Zvia Breznitz

**Affiliations:** The Edmond J. Safra Brain Research Center for the Study of Learning Disabilities, Faculty of Education, University of Haifa, Haifa, Israel; University of Sydney, Australia

## Abstract

**Background:**

Based on the relationship between working memory and error detection, we investigated the capacity of adult dyslexic readers' working memory to change as a result of training, and the impact of training on the error detection mechanism.

**Methodology:**

27 dyslexics and 34 controls, all university students, participated in the study. ERP methodology and behavioral measures were employed prior to, immediately after, and 6 months after training. The CogniFit Personal Coach Program, which consists of 24 sessions of direct training of working memory skills, was used.

**Findings:**

Both groups of readers gained from the training program but the dyslexic readers gained significantly more. In the dyslexic group, digit span increased from 9.84±3.15 to 10.79±3.03. Working memory training significantly increased the number of words per minute read correctly by 14.73%. Adult brain activity changed as a result of training, evidenced by an increase in both working memory capacity and the amplitude of the Error-related Negativity (ERN) component (24.71%). When ERN amplitudes increased, the percentage of errors on the Sternberg tests decreased.

**Conclusions:**

We suggest that by expanding the working memory capacity, larger units of information are retained in the system, enabling more effective error detection. The crucial functioning of the central-executive as a sub-component of the working memory is also discussed.

## Introduction

Dyslexia is a cognitive disorder characterized by a high rate of decoding errors when reading printed materials [1]–[4]. Studies have suggested that cognitive errors are monitored by an error detection mechanism that can be identified during task performance by two Event-Related Potentials (ERP) on an on-going electroencephalogram (EEG): error- related negativity (ERN) and correct-related negativity (CRN) [5]–[7]. The ERN and the CRN components are evoked 0–160 ms following an erroneous or a correct response, respectively [5]–[7].

In view of the high frequency of errors among dyslexics, we undertook in a previous study to determine whether the error detection mechanism operates in them during reading [8]. The results of the study, which investigated the brain activity of dyslexic university students during performance of various linguistic tasks and while committing reading errors, indicated the existence of the error detection mechanism during reading. However, the strength of its activity among dyslexics differed from that of controls: while committing an error, dyslexics displayed significantly lower ERN amplitudes than controls. We suggested that this low activity of the error detection mechanism might prevent dyslexics from becoming aware of their errors and learning from them.

A number of theories have been raised to explain the ERN evocation, including the mismatch theory [5], the negative feedback signal theory [9], the conflict theory [10], the learning reinforcement theory [11], and the characteristics of the working memory system [12]–[14].

Horowitz-Kraus and Breznitz (submitted) suggested that activation of the error detection mechanism is affected by characteristics of the working memory system [12]–[14]. The limited capacity and rapid decay of information [15] in the working memory affects the short-term consolidation of information in long-term memory [13]. This imposes limitations on the retrieval of correct responses from long-term memory [14] that affect the activation of the error monitoring system when processing information [12]. Horowitz-Kraus and Breznitz's results further indicated that when the linguistic task becomes more complex and imposes an additional load on the working memory, the ERN amplitude decreases.

Several studies showed dyslexic readers to have significantly lower working memory capacity compared to controls [16]–[26]. In general, working memory is a sub-component of the information processing system, which has been defined as capacity-limited [27]. Yet working memory enables short-term storage of information, which is made available for processing and integration [27]. The working memory is composed of three sub-components: the central executive, which allocates attention resources for a specific task and is responsible for processing the information stored within working memory [28]; the phonological loop, which is a verbal information processor; and the visuo-spatial sketchpad, which is responsible for visuo-spatial information processing [27]. A fourth sub-system has also been suggested – the episodic buffer, which stores information from the other sub-components in long-term memory [29].

Accumulated data has pointed to the human brain's plasticity and ability to adapt to change [30]–[33]. Changes can occur not only in the young brain [34], but in the adult as well ([30], [31], [35], [36], see especially [37]). A critical factor for triggering brain plasticity is training [e.g.], [ 33], [35], [38]–[42]. In recent years, intervention programs have been shown to change behavior and brain activity of dyslexic readers [43], [44]. The increase in the capacity of working memory following training was evidenced by increased cortical activity in pre-frontal and parietal areas [45]. A recent study [46] investigated the ability of working memory capacity to expand following memory training using the CogniFit Personal Coach (CPC) program [47] in dyslexics. Not only did their working memory expand significantly after training, their reading performance improved and brain activity changed significantly as evidenced by the early latency and higher amplitude of the P300 ERP component.

Based on these findings, we set out to examine the relationship between working memory capacity and ERN in dyslexics and controls. This entailed an attempt to replicate the earlier findings about the potential to enhance working memory by specific cognitive training, and to assess the impact of such training on the error detection system. We hypothesized that training working memory would increase both the working memory and ERN amplitudes of dyslexics. Electrophysiological measures were obtained using the ERP methodology.

## Methods

### Participants

61 university students (27 dyslexic and 34 controls) participated in the study. Both groups were matched for chronological age (25.2±3.5 years) and were within normal nonverbal IQ range as measured by the Raven Standard Progressive Matrices [48] [T(2,59) = 3.36, *P*>.05 for the Raven matrix test: X = 109±2.13 for the dyslexics and X = 112±1.11 for the controls]. All were native Hebrew speakers from a middle-class background. All subjects were right-handed, displayed normal or corrected-to-normal vision in both eyes, and were screened for normal hearing. None of the participants had a history of neurological or emotional disorders, or attention deficit as measured by the D2 test [49] [T(2,59) = 3.39, *P*>.05, for the attention D2 test: X = 8.76±1.45, for the dyslexic readers and X = 8.88±1.23 for the controls].

All subjects gave their informed consent prior to inclusion in the study, and all were paid volunteers. The dyslexic readers were recruited through the Student Support Service of the University of Haifa, which assists students with learning disabilities. They were diagnosed as dyslexic during childhood and classified as impaired readers by the Student Support Service. The controls were recruited by notices posted on bulletin boards on the University campus. The experiment was approved by the University of Haifa ethics committee.

### Measures

#### Behavioral and Experimental measures

The experimental procedure, and behavioral and experimental measures are summarized in Table 1.

**Table 1:** Experimental procedure, and behavioral and experimental measures.

		Test 1	Training	Test 2	Test 3
**Time**		Prior to training		Immediately after training	6 months after training
**Behavioral measures**	**Reading measures**	A. Decoding- One minute words/pseudowords test* [1].	The training program lasted six weeks*****.	A. One minute words/pseudowords test.	A. One minute words/pseudowords test.
		B. Fluency- Oral reading test** [50].			B. Oral reading test.
		C. Comprehension- Silent reading test containing 15 closed questions [51].		C. Comprehension test.	C. Comprehension test.
**Experimental measures**	**Memory measures**	A. Short term memory and capacity- digit span subtest from the WAIS-III [52].		A. Digit span test	A. Digit span test
		B. Verbal Working memory- The Opposites test*** [1].		B. Opposites test	B. Opposites test
	**Compute-rized CPC Memory** sub-tests [47]. Both accuracy and rate measures****.	A. Short term visual memory -Recalling digits (forward and backward) which were presented visually on the computer screen.		A. Short term visual memory	A. Short term visual memory
		B. Short term auditory memory-Recalling digits (forward and backward) presented via headsets to the auditory modality.		B. Short test auditory memory	B. Short test auditory memory
		C. Cross modality short term memory recall- Simultaneously presentation of digits to both modalities.		C. Cross modality short term recall	C. Cross modality short term recall
**Electrophysiolo-gical measures**		Sternberg task [53].		Sternberg task	Sternberg task

* In the One minute words/pseudowords test, the reader was asked to read as fast and as accurate as he/she could two separate lists of words and pseudowords in Hebrew in one minute.

** The orally connected text contained 264 words. The texts were taken from the Reading Test section of the Israeli Psychometric SAT [50]. A measure of word per second was calculated for each test.

*** This test contained an increased number of adjectives and the subject was asked to name their opposites in the same order they were introduced. The test examined accuracy when producing the correct adjectives by their order.

**** These tests were given to the subjects in the form of games rather than formal tests. Each task had detailed instructions and a short practice session before the actual test. If the user did not fully understand the task, the computer presented a reminder of the task's rules. The program ensured that the user understood the task and then started measuring his/her performance. Each of the memory measures for each subject was later compared to the database of age-matched normal population measurements [47], yielding the subject's relative memory skill performance. According to the subject's baseline evaluation prior to the experiment, the training program offered him/her the most suitable training program.

***** The training program lasted six weeks with four sessions per week (approximately 20 minutes each) for a total of 24 intervention sessions for each subject (see Methods section).

#### Electrophysiological Measures

In order to comply with the ERP methodology, which requires a high number of repetitive trials, brain activity during working memory task performance was examined using a Sternberg task [53]. This task is commonly used in behavioral and electrophysiological studies for studying processing in working memory (modeled after [54]). The Sternberg task consisted of a five-digit series presented visually on a computer screen. Each digit was presented for 500 milliseconds (ms) with an ISI of 700 ms. At the end of each series, a line of stars was presented for 1000 ms, followed by 300 ms of a blank screen and then a probe presented for 500 ms. The subjects were instructed to determine whether or not the probe appeared in the series of digits presented, pressing the right joystick button when the probe appeared and the left when it did not. The next series appeared after 2500 ms. The task was comprised of 60 experimental series of numbers divided into two separate sections, each presenting 30 items.

#### Overt performance

Mean reaction time (RT) was calculated separately for all correct and error responses. Only responses between 300 ms and 1700 ms after target onset were included in the mean RT.

### Training materials - the CPC program [47]

Auditory, visual, and cross-modality working memory skills were trained using the *CPC Computerized Cognitive Program* [47]. This program includes a baseline cognitive assessment (T1) that allows the training program to be individualized for each subject. Normative data from a large database of previous users define the initial challenge level of each of the memory training tasks used. Each training session includes a mixture of visual, auditory and cross-modality tasks aimed at training working memory capacity. Each of the three tasks has three levels of difficulty – easy, moderate and difficult. The training program and the level of the complexity within each domain were created for each subject according to a personal ‘Scheduled Training Option’ [47] that was based on the subject's performance on the baseline assessment. The level of challenge was further readjusted after each training session according to the subject's progress. Each training session took 15–20 minutes.

At the beginning of each task, the user read a description of the main cognitive skills that were being trained in that task. At the end of each task's daily training, the user could examine his/her performance on graphs that described his/her progress.

### Examples of the cognitive tasks used in the CPC program:

#### Auditory Memory task

The subject was introduced to sequential sounds of different lengths by various musical instruments. Later, the subject was asked to identify the instruments' sequence by their sound, and to identify some tempo patterns. The length of the sequence varied from task to task.

#### Visual Memory task

Windows in a house on the screen opened in a certain sequence. The subject was asked to follow the exact sequence in which the windows opened. The length of the sequence varied from task to task.

#### Cross modality memory task

Pictures and sounds of objects were presented to the subject sequentially. The subject then had to recognize the objects from an array including irrelevant probes, and recall whether it was presented visually or auditorily. The length of the sequence varied according to the training stage.

An important feature of the program was the Personal Coach. The personal coach offered insights and advice based on data from several sessions. For example, if a subject exhibited good visual memory abilities but poor auditory memory abilities, the program would comment on the skill difference and advise the subject to pay special attention to auditory memory tasks.

### Apparatus

**Figure 1:**
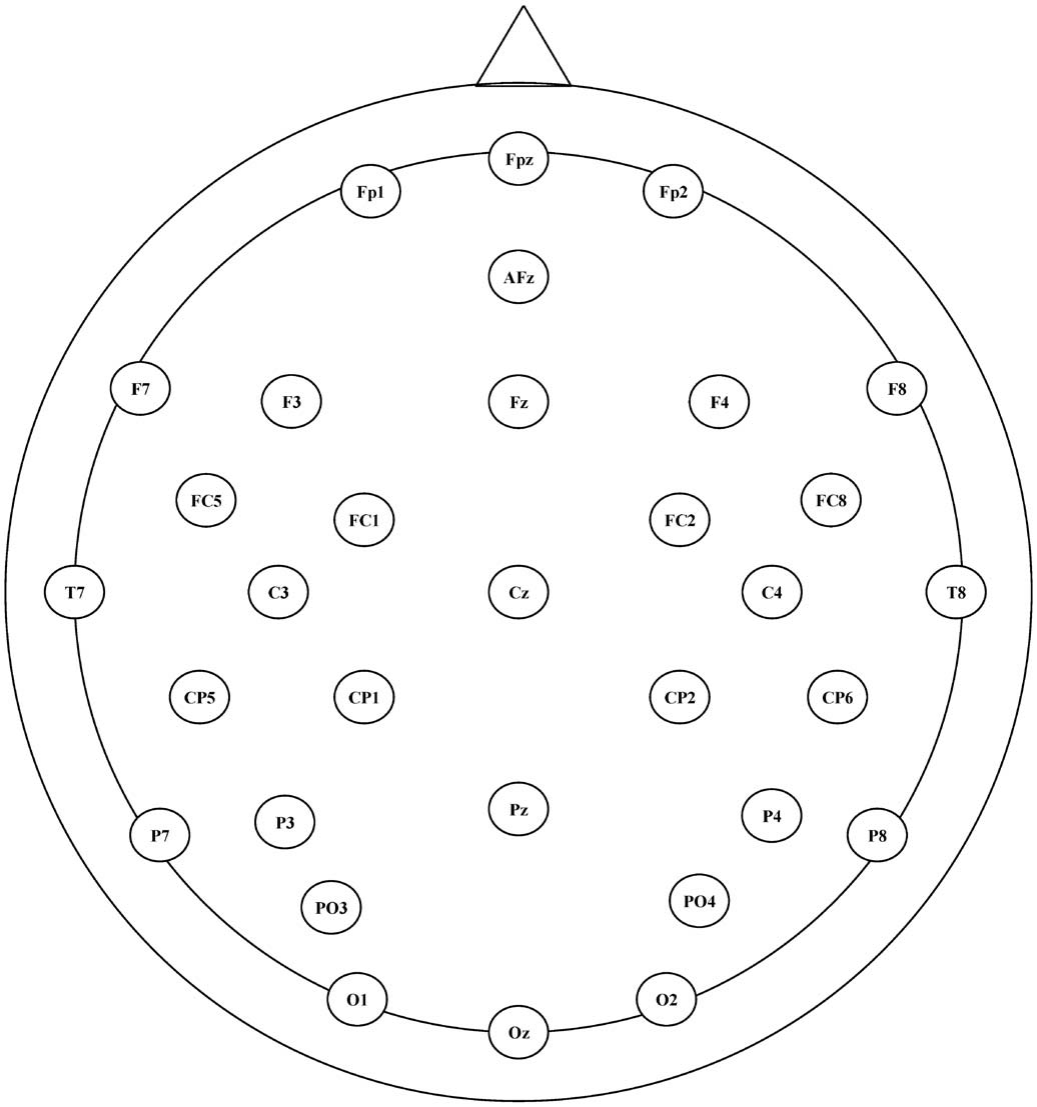
Distribution of electrodes on the scalp.

The EEG was recorded continuously via 31 electrodes mounted on a custom-made cup (Bio-logic Ltd.), according to the international 10/20 system [55] (see Figure 1), sampled at a rate of 256 Hz with an analog band pass filter of 0.1 to 70 Hz and 12-bit A/D converter and stored for off-line analysis. An electro-oculogram (EOG) was recorded with an electrode extension (Oz) that was located under the left eye. An electrode on the chin served as reference for both EEG and EOG recordings. A ground electrode was placed on the left mastoid. All electrodes were maintained at an impedance of 5 KΩ or less.

The EEG was corrected for horizontal and vertical eye movements using Gratton, Coles and Donchin's algorithm [56] as implemented in the Vision Analyzer (version 1.05) program, and filtered with a 25 Hz filter. ERPs resulted from averaging epochs starting 100 ms before and ending 400 ms after response onset. The averaging record was separated into correct and error records. Each average record contained a recording of the probe appeared in the third place in the sequence of five digits, and records that did not contain the probe. All single trials were inspected visually and were free of residual artifacts. The baseline for the ERN and CRN components was -100 ms–0 ms pre-response. The amplitudes of the components were defined as the largest negative peak in the 30–150 ms interval post-response onset for the ERN and CRN.

### Research design and procedure

General ability, attention, reading, word recognition, and memory measures were administered prior to the experiment in order to verify that both dyslexics and controls met the criteria of dyslexia and regular reading (standard score of ≤−1.5 on a standard reading test in Hebrew, see [57]). In general, each behavioral testing session lasted approximately 1.5 hours (see Table 1).

During the experiment, the subject was seated in an isolated sound-attenuated room. No interaction took place between the subject and experimenter during the task. The stimuli were presented on a computer screen located 1.5 m in front of the subject and all the auditory stimuli were presented via headsets from the computer at a frequency of 2000 Hz.

## Results

### Behavioral measures

Baseline tests of reading skills were performed prior to the experiment to verify the assignment of subjects to the research groups. T-test analyses revealed no significant group differences on any of the general abilities parameters.

Significant differences were found between the two groups on two out of three reading measures. The dyslexics read significantly fewer words and pseudowords in one minute and read at a significantly slower rate compared to the controls. However, in line with previous studies [1], no group differences were found for the reading comprehension score (Table 2, T1, results for groups A and B).

**Table 2:** A comparison of dyslexics and controls on reading measures for all tests.

		Test time 1		Test time 2		Test time 3			
**Reading measure**		Dyslexics M(SD)(A)	Controls M(SD) (B)	Dyslexics M(SD) (C)	ControlsM(SD) (D)	Dyslexics M(SD) (E)	Controls M(SD) (F)	Univari-ate F	T value
One minute test for words	Accuracy	71.19 (16.31)	117.27 (17.98)	79.23 (18.85)	120.88 (18.11)	81.68 (20.44)	122.63 (16.79)	B>A	(−10.173***)
								D>C	(−8.672***)
								F>E	(−8.375***)
One minute test for pseudowords	Accuracy	35.57 (10.19)	64.09 (11.63)	37.5 (11.17)	68.73 (10.61)	40.88 (13.72)	70.93 (10.27)	B>A	(−9.864 ***)
								D>C	(−11.039***)
								F>E	(−9.545***)
Silent reading comprehension	Accuracy	13.33 (1)	13.84 (1.27)	14.16 (.68)	13.97 (.959)	13.04 (1.33)	13.48 (1.17)	B = A	(−1.638)
								C = D	(.815)
								F = E	(−1.327)
Oral reading	Speed	.616 (.21)	.41 (.04)	.6 (.18)	.4 (.051)	.53 (.18)	.48 (.12)	A>B	(4.833***)
								C>D	(5.136***)
								E>F	(1.142)

*P<.05; **P<.01; ***P<.001.

Mean (M) and Standard deviation (SD) for accuracy (in percentage) and speed (in milliseconds) of reading measures in dyslexics and controls for tests 1, 2 and 3.

### Experimental tasks

In order to verify the significant differences between the two groups prior to, immediately following, and six months post-training, (2×3) Repeated Measures factorial Analyses of Variance (RM-ANOVAs) {[Group (Dyslexics × Controls)] × Test time (T1×T2×T3)]} were conducted separately for each of the experimental measures.

A. Digit-span - Wechsler-III subtest: A significant main effect of Group [*F*(2,59) = 8.206, *P*<.01] was found, indicating a higher digit-span score in the control group compared to dyslexics. A significant main effect of Test Time was found [*F*(3,58) = 6.911, *P*<.001], with digit-span for both groups higher at T3. A Group × Test Time interaction was found [*F*(5,56) = 5.146, *P*<.01]. The interaction stemmed from the dyslexics having the greatest differences between T1 and T3. Means and SDs are presented in Table 3.

**Table 3:** A comparison of dyslexics and controls on memory measures for all tests.

		Test time 1		Test time 2		Test time 3			
**Measures**	**Measure**	DyslexicsM(SD)(A)	ControlsM(SD)(B)	DyslexicsM(SD)(C)	ControlsM(SD)(D)	DyslexicsM(SD)(E)	Controls M(SD)(F)	Univariate F	T value
Behavioral	Digit span	9.84 (3.15)	11.75 (2.82)	9.72 (2.8)	12.07 (2.77)	10.79 (3.03)	12.42 (3.52)	B>A	(−2.448**)
								D>C	(−4.421***)
								F>E	(−2.159*)
	Working memory	−.58 (.61)	.06 (.86)	−.48 (.506)	.28 (.89)	−.42 (.69)	.61 (.805)	B>A	(−3.078**)
								D>C	(−4.129***)
								F>E	(−5.112***)
CPC	Auditory memory	−.09 (.53)	.15 (.55)	.2 (.63)	.3 (.52)	.2 (.63)	.3 (.52)	B>A	(−1.792)
								D>C	(−.661)
								F>E	(−.661)
	Visual memory	−.04 (.68)	.33 (.84)	.05 (.91)	.77 (.88)	.49 (.62)	.74 (.79)	B>A	(−1.94*)
								D>C	(−3.166**)
								F>E	(−1.388)
	Cross modalities Memory	−.05 (.85)	−.11 (.77)	.39 (1.04)	.34 (.74)	.34 (.47)	.74 (.77)	A>B	(.3)
								C>D	(.23)
								F>E	(−2.67**)

*P<.05; **P<.01; ***P<.001.

Means (M) and standard deviation (SD) of dyslexics versus controls on the memory measures: standard scores for behavioral measures (digit-span and working memory) and for CogniFit Personal Coach (CPC).

*B.* Working-memory - Opposites: A significant main effect was found for Group [*F*(2,59) = 17.511, *P*<.000], with a higher score for controls compared to dyslexic readers, and for Test Time [*F*(3,58) = 4.38, *P*<.05], with the highest score for both groups at T3. In addition, a Group × Test Time interaction was found [*F*(5,56) = 6.055, *P*<.01], stemming from the highest differences between T1 and T3 in the control group. Means and SDs are presented in Table 3.

C. Sternberg task: A (2×2×3) RM-Manova {[Group (Dyslexics × Controls)] × [Response (Correct × Errors)] × [Test Time (T1×T2×T3)]} was obtained for accuracy and reaction time separately.

*Accuracy*: Results indicated a significant main effect of Group [*F*(2,59) = 16.871, *P*<.000], Response [*F*(2,59) = 5.393, *P*<.01], and Test Time [*F*(7,54) = 6.521, *P*<.01]. Controls were more accurate than dyslexic readers. Positive correct responses were more accurate than negative correct responses. Of some interest was the finding that accuracy was highest at T3. No significant Group × Response interactions were obtained. Means and SDs are presented in Table 4.

**Table 4:** Accuracy rate and reaction times for the experimental measures.

		Test time 1		Test time 2		Test time 3			
**Measures**	**Type of response**	DyslexicsM(SD) (A)	ControlsM(SD) (B)	DyslexicsM(SD) (C)	ControlsM(SD) (D)	Dyslexics M(SD) (E)	Controls M(SD) (F)	Univariate F	T value
Accuracy rate	Correct	79.39 (18.67)	90.2 (13.53)	87.36 (7.48)	94.39 (9.55)	89.37 (10.1)	94.49 (6.77)	B>A	(2.828**)
								D>C	(6.201***)
								F>E	(4.943***)
	Error	11.64 (8.74)	3.28 (3.63)	10.62 (6.05)	4.14 (4.16)	8.4 (6.58)	4.14 (4.43)	A>B	(2.283*)
								C>D	(2.866**)
								E>F	(.579)
Reaction time	Correct	1186.88 (361.09)	964.71 (231.6)	1093.73 (120.97)	860.94 (152.09)	1107.68 (195.8)	848.68 (194.95)	A>B	(−2.534*)
								C>D	(−2.996**)
								E>F	(−2.291*)
	Error	1137.96 (516.31)	831.72 (487.9)	988.13 (346.95)	742.72 (297.65)	998.18 (359.35)	923.84 (548.78)	A>B	(4.418***)
								C>D	(4.791***)
								E>F	(2.75**)

*P<.05; **P<.01; ***P<.001.

Means (M) and standard deviation (SD) of dyslexics versus controls on the Sternberg task: accuracy rate (in percentage) and reaction times (in milliseconds).

*Reaction time (RT*): A significant main effect of Group [*F*(2,59) = 4.566, *P*<.05] and Response [*F*(2,59) = 25.129, *P*<.000] was found, indicating longer RT for the dyslexics compared to controls and longer RT for correct compared to error responses for both groups. In addition, RT was shorter on T2 for both groups. No significant interactions were obtained. Means and SDs are presented in Table 4.

D. CPC memory test [47]: A (2×3×3) RM-Manova {[Group (Dyslexics × Controls)] × [Memory test (Visual × Auditory × Cross Modal)] × [Test Time (T1×T2×T3)]} was obtained for the CPC memory measures. Main effects of Group [*F*(1,60) = 7.451, *P*<.01], Memory [*F*(2,59) = 5.22, *P*<.01], and Test time [*F*(2,59) = 23.184, *P*<.000] were found. The dyslexics had lower memory scores than controls. A Group × Test time × Memory interaction [*F*(4,57) = 4.637, *P*<.03] indicated an increase in the cross-modal memory score after training for both groups. However, at T3 the cross-modal score increased among the controls and decreased among the dyslexics. Means and SDs are presented in Table 3.

**The above results replicate earlier findings of the beneficial effects of cognitive training on working memory, and paves the way for testing its impact on indicators of brain activity.**

### Electrophysiological measures

A (2×2×3) RM-Manova {[Group (Dyslexics × Controls)] × [Response (Correct × Errors)] × [Test Time (T1×T2×T3)]} was conducted to verify significant differences between dyslexics and controls for correct and erroneous responses before, immediately following, and six months post-training. Since the response-locked ERN and CRN components were observed at the Cz electrode (in accordance with Russeler, Kuhlicke and Munte, see [58]), measurements of amplitude from this electrode were used in the analyses.

### The ERN and CRN components

#### Amplitude

**Figure 2:**
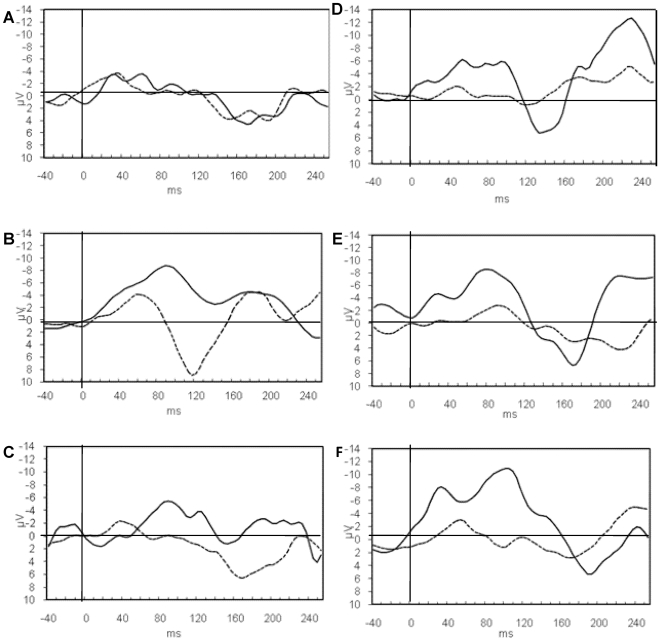
ERN and CRN amplitudes in tests 1, 2 and 3 in dyslexics and controls. A grand average for the ERN-CRN (response-locked) components for correct response (CRN) and for error response (ERN), represented by the dashed and the solid lines, respectively, for tests 1 (A,D), 2 (B,E) and 3 (C,F) in dyslexics (left column, A,B,C) and controls (right column, D,E,F) at the Cz electrode. The ERN is seen between 30 and 150 ms after response denoted by the vertical line at time 0. Note that the negative Y axis is plotted up.

A significant main effect of Group [*F*(2,59) = 4.792, *P*<.05], Test [*F*(3,58) = 10.511, *P* <.000] and Response [*F*(2,59)  = 410.149, *P*<.000] was found, indicating a higher amplitude for the controls compared to the dyslexic readers. However, this was due entirely to higher amplitudes for errors, which were highest for T2 and lowest for T1 in both groups. A Group × Response × Test Time interaction [*F*(7,54) = 25.094, *P*<.000] indicated the highest gap between the ERN and CRN amplitude sizes at T2 and the lowest at T1 in the dyslexic readers. See Table 5 and Figure 2 for means and SDs.

**Table 5:** Amplitudes and latencies of response-locked ERP components.

		Test time 1		Test time 2		Test time 3			
**Measure**	**Type of response**	Dyslexics M(SD) (A)	Controls M(SD) (B)	Dyslexics M(SD) (C)	Controls M(SD) (D)	Dyslexics M(SD) (E)	Controls M(SD) (F)	Univariate F	T value
Amplitude	Correct	−5.65 (1.86)	−4.75 (1.95)	−5.66 (1.93)	−4.56 (1.45)	−5.57 (1.84)	−5.9 (1.9)	A<B	(−1.78)
								C<D	(−1.93)
								F<E	(.635)
	Error	−6.15 (1.43)	−9.9 (1.49)	−10.91 (2.41)	−9.16 (1.37)	−7.67 (1.66)	−10.19 (1.04)	B>A	(9.63***)
								C>D	(−3.295***)
								E>F	(6.616**)
Latency	Correct	59.49 (18.17)	60.31 (16.22)	65.97 (17.69)	67.66 (24.21)	77.45 (19.16)	73.1 (23.52)	B>A	(−.181)
								D>C	(−.3)
								E>F	(.743)
	Error	61.49 (8.29)	66.74 (4.61)	78.24 (9.45)	73.26 (7.83)	78.84 (7.41)	78.26 (6.92)	B>A	(−2.94**)
								C>D	(2.138*)
								E>F	(.299)

*P<.05; **P<.01; ***P<.001.

Amplitudes (in µV) and latencies (in milliseconds) of response-locked ERP (ERN and CRN) components at the Cz electrode for dyslexics and controls.

**The above confirms our central hypothesis that training working memory enhances the ERN of dyslexics.**

### The correlation between working memory capacity and the amplitude of ERN

Moderate correlations were found between the amplitude of ERN and the capacity of working memory. Prior to training (T1), the correlation was r = .28, *P*<.05 for the controls and r = .45 (*P*<.001) for the dyslexic readers. Immediately after training (T2), the correlations remained essentially unchanged: r = .26 and r = .44, respectively. At the long post-training time (T3), the correlations increased to r = .41and r = .50. Results indicated that **the larger the working memory capacity, the higher the ERN amplitude. This relationship between working memory capacity and ERN amplitude was obtained at all test times and was higher for the dyslexic readers.**

## Discussion

The objective measures used in this study support previous results indicating that even compensated dyslexic university students continue to exhibit a high rate of word reading errors, dysfluent reading, and lower capacity of working memory compared to age-matched controls (see [1] for review). The data also confirm previous findings [8] of lower ERN amplitudes in dyslexic subjects compared to controls. Despite being compensated adult dyslexic university students who had been exposed to printed materials for years and supported by remedial reading programs, their error detection system was functioning at a suboptimal level. It is conceivable that both the decoding inaccuracy and dysfluency in word reading that are at the core of the definition of dyslexia [2] are due, at least in part, to an inadequately functioning error detection monitoring system. This, in turn, reduces awareness of decoding mistakes during reading, making their subsequent correction more difficult.

The modus operandi of the error detection system remains elusive. We argue here that working memory capacity plays an important role in its functioning: that is, a larger working memory capacity leads to a higher ERN amplitude. This has been documented in the literature in both a between-individuals comparison and in the effects of increasing working memory by cognitive training (e.g., [45], ). While such a relationship between working memory and ERN has been suggested in previous studies [12] [14], the subjects in the current study – adult dyslexic university students who prior to the training exhibited not only lower reading scores, but also a limited capacity of the working memory system and a lower amplitude of ERN - make the findings especially compelling.

The observed increase in reading performance following working memory training and its concomitant ERN enhancement has implications for improving the design of interventions with dyslexics. At the same time, it is obvious that the causal links between working memory, ERN and performance are complex. In an effort to better understand this process, we partialled out working memory from the formula and calculated the correlation between accuracy on the Sternberg task and ERN amplitude. The correlation at T1 was found to be r = 34 (*P*<05), essentially the same as that found between them without partialling out working memory (r = .33). This suggests that the contributions of working memory and the error detection system to performance are partially independent of each other. To further complicate matters, it is conceivable that working memory enhances the effectiveness of the error detection system, but once in place it operates at least to some extent independently of it.

How do the changes in working memory capacity following training affect the error detection mechanism? A possible explanation may lie in the central executive (CE) working memory sub-system. According to the literature, the CE is responsible for allocating processing resources and for increasing the amount of information stored in the phonological loop and the visual-sketchpad systems. If the storage requirements for processing are larger than the sub-system's capacity, the CE allocates processing resources and increases storage capacity in the other two sub-systems [27], [59]. Storage capacity can be measured; the literature states that the appropriate range is 7±2 items [52], [60]. It is possible that the working memory training program used in the present study not only affected the storage capacities of the phonological loop (as observed in the working memory of opposites, digit span, and CPC auditory tasks) and the visual sketchpad (as observed in the Sternberg task, which was presented visually to the subjects on the computer screen, and in the CPC visual tasks), but also of the CE. Initial evidence for this claim comes from our finding of an increase in accuracy in the cross-modality tasks following training in both groups. It follows that cross-modality tasks are processed in the CE. By extension, word reading may also be processed in the CE, as it requires the integration of graphemes and phonemes. Performance on tasks that require pure word identification skills were also enhanced following training in both our groups, more prominent among the dyslexics (details in paragraph two of Results). This implies that direct working memory training enables larger patterns to be retained and monitored by the error monitoring system, thereby reducing the possibility of overloading the CE (see [12]).

The mismatch theory claims that the ERN component is evoked following the execution of an error, and is the result of a comparison and discrepancy between the desired and actual response representations [5]. Moreover, the ERN amplitude is correlated with the degree of incompatibility between representations [61]. It can be hypothesized that as storage capacity increases, more representations are stored in the system, including the “desired” response, and error negativity is higher. Accordingly, thanks to the existence of the representations the conflict is also lower, and according to the conflict theory so is the CRN amplitude.

An abundance of data has pointed to the human brain's plasticity and ability to adapt to change [30] [33]. Nevertheless, the persistence of reading deficits into adulthood despite the accumulation of experience in reading has been attributed in part to the closing of ‘critical’, early-life time windows of increased brain plasticity [62] [64] in which neuronal systems are particularly susceptible to shaping by experience. These findings led to the conclusion that remedial interventions would be less effective in adults [62]. However, evidence is accumulating in support of the notion that rather than having less effective skill-learning (‘how to’, procedural) or memory consolidation processes per se, adults may be more selective in terms of procedural memory consolidation compared to children (e.g., [37]). This was borne out in our study where both groups of adults showed an increase in the working memory capacity after training. This may account, in part, for the discrepancy between a simple notion of maturational windows of opportunity in the acquisition of skills on one hand, and the accumulating evidence for very effective skill-learning in adults on the other (e.g., [65]). In can be claimed that the discrepancy between our and others' findings might by attributable to differences in methodology: the skills in our program might relate more closely to the effectiveness of working memory; and our study entailed more precise measurement of the effectiveness of the training program

It is clear that the capacity of the working memory system is limited [60]. Within this limit, dyslexics have lower working memory storage capacity than do controls (e.g., [16], [23]). The fact that the dyslexics' working memory capacity improved following training more than that of the controls may be because the initial gap between their ability and their performance is wider and they have much more to gain from training. It is also possible that their working memory storage capacity is still flexible and less stable. This notion might also explain why the largest gains in working memory function among dyslexic readers were made immediately following the training program (T2). This group benefit was also evident at T3, but at a lower level than at T2. It is conceivable that in order to maintain working memory skills, an on-going, low-scale training is needed for this group of readers.

Conversely, the regular readers gained the most in various working memory skills at the long post-training time (T3, after six months). It can be hypothesized that the changes in brain activity among regular readers immediately following training are initially at the level of the stimulus perception, as their performance is basically automatic (see the amplitudes of the CRN component) and there is almost no need for them to activate the error detection monitoring system (as there is a low amount of errors). However, because their current system resources are sufficient for adequate functioning, they need longer to consolidate the skill in which they were trained [33], [39], [42]. Further studies are required to examine this concept in depth.
